# The Role of Skin Innervation for Assessment of Neurological Involvement in Disorders: A Review

**DOI:** 10.3390/brainsci15121254

**Published:** 2025-11-21

**Authors:** Alessandro Furia, Cosmanna Ragucci, Rocco Liguori, Giovanni Rizzo, Veria Vacchiano, Maria Pia Giannoccaro, Vincenzo Angelo Donadio

**Affiliations:** 1 Dipartimento di Scienze Biomediche e Neuromotorie, Università di Bologna, 40126 Bologna, Italy; alessandro.furia3@unibo.it (A.F.);; 2 IRCCS Istituto delle Scienze Neurologiche di Bologna, UOC Clinica Neurologica, 40139 Bologna, Italy

**Keywords:** skin biopsy, nerve fibers, synucleinopathy, Fabry, small fiber neuropathy

## Abstract

Skin biopsy is an affordable, minimally invasive technique that provides direct access to peripheral neural structures for in vivo evaluation of cutaneous nerve pathology, with relevance to both peripheral and central nervous system disorders. Skin biopsy allows the assessment of somatic and autonomic fibers as well as their innervated structures, representing the gold standard for the diagnosis of Small-Fiber Neuropathy. Nonetheless, the assessment of autonomic fibers remains challenging, as the patterns of sympathetic and parasympathetic skin innervation have not yet been fully elucidated, and the intricate organization of effector structures (e.g., arrector pilorum muscles, sweat glands, blood vessels) poses methodological difficulties. Beyond small-fiber evaluation, skin biopsy allows the detection of disease-specific deposits of abnormally accumulating proteins in a broad spectrum of clinical entities. It has proven highly accurate in detecting synucleinopathies in vivo, with near-complete specificity in the discrimination of affected patients from healthy controls and from alternative neurodegenerative disorders. Furthermore, the pattern of α-synuclein deposition serves to differentiate Lewy body from non-Lewy body synucleinopathies, thereby distinguishing disorders with similar clinical manifestations but distinct physiopathology and prognostic implications. In this narrative review, we outline the current indications for skin biopsy in the evaluation of diverse neurological disorders and address the main methodological aspects of the technique.

## 1. Introduction

The skin constitutes the largest organ of the human body: its innervation is complex and reflects the multitude of different structures found from the dermis to the epidermis.

Aside from thickly myelinated, large nerve fibers (e.g., Aβ), which innervate dermal structures responsible for proprioception, the nervous system also provides the skin with both somatic and autonomic nerve fibers of small caliber, namely thinly myelinated Aδ and unmyelinated C fibers [1]. Somatic fibers ascend the dermal–epidermal junction, terminating as free nerve endings which convey pain and temperature perception. On the other hand, autonomic nerve fibers innervate different skin components, such as blood vessels, sweat glands, and arrector pilorum muscles, to allow for different processes regulating body homeostasis [2].

Historically, pathological studies were available for large nerve fibers only, more specifically via peripheral nerve biopsy: this constitutes an invasive procedure on sensory (e.g., sural nerve) and, less commonly, motor nerves, which nonetheless provides key etiologic information on peripheral neuropathies. The demonstration that small nerve fibers could be stained and visualized allowed for a new window to be opened into the nervous system by exploiting skin biopsy, an affordable, rapid, and lowly invasive method when compared to other methods of sampling for the nervous system (e.g., lumbar puncture).

More particularly, by exploiting the pan-axonal marker Protein Gene Product 9.5 (PGP9.5), somatic and autonomic nerve fibers of the skin can be marked and observed, and the innervated skin structures evaluated [3]. Among its various applications, skin biopsy has become a staple technique for the diagnosis of Small-Fiber Neuropathy, in which intraepidermal nerve fiber density is reduced at the distal leg site [1].

Nonetheless, recent staining techniques have increasingly aimed to explore additional targets of small nerve fibers. This applies particularly to the autonomic system, whose exact pattern of innervation is still in discovery, but also to pathological deposits which can be found in neurological and systemic disorders, such as Fabry Disease, synucleinopathies, and Cerebral Autosomal Dominant Arteriopathy with Subcortical Infarcts and Leukoencephalopathy (CADASIL).

Moreover, the staining of larger myelinated skin nerve fibers has allowed for the study of Large-Fiber Neuropathies, such as in inherited forms, making skin biopsy a potentially feasible and reproducible method of extracting biomarkers of disease in these entities, too.

The following narrative review was devised to highlight the advantages of skin biopsy in neurological diseases. It is acknowledged that a narrative approach is less robust in method than a systematic revision of the literature, but this approach was chosen in consideration of the many and multifaceted groups of different neurological disorders, with the aim of enhancing readability while still offering a clear enough picture of the state of the art.

## 2. Neuropathies

### 2.1. Small-Fiber Neuropathy

Small-Fiber Neuropathy (SFN) represents a set of disorders in which small nerve fibers are damaged, leading more specifically to alterations in nociception (e.g., neuropathic pain), thermoception, and, less commonly, dysautonomia.

While skin biopsy cannot provide in vivo information, especially in cases of functional-only alterations of small nerve fibers, it allows for reliable qualitative and quantitative assessment of skin innervation. It provides reliable and reproducible pathological data, such as intraepidermal nerve fiber density (IENFD), which has been shown to have high intra- and interlaboratory reliability and reproducibility.

This aspect is particularly relevant due to the vast array of etiologies that have been linked to SFN, comprising metabolic, infectious, autoimmune, drug-induced/toxic inherited, and idiopathic ones [4,5,6,7,8,9]. In all of these cases, skin biopsy can be used to demonstrate reduced IENFD, consistent with SFN, and in some cases morphological alterations of small nerve fibers consistent with prodromal stages preceding full-fledged neuropathy [4,10].

More specifically, this was the case for two widespread disorders associated with painful symptomatology, i.e., HIV infection and diabetes, in which SFN has been regarded as an initial phase of neuropathy, potentially progressing to the more commonly recognized large-fiber polyneuropathy [4,11]. Among other studied causes, the COVID19 pandemic has been linked to the development of chronic, long-lasting symptomatology referred to as “long COVID”: when this is underlined by painful and/or dysautonomic symptomatology, the role of SFN has been explored [12].

Skin innervation is assessed by immunohistochemical techniques, in which primary antibodies bind to the target antigen and are then bound to secondary antibodies which can then be visualized. Two main methods have emerged in analysis of small nerve fibers: bright-field immunohistochemistry and indirect immunofluorescence [3].

The former employs a peroxidase reacting with the secondary antibody to produce precipitates which can then be visualized with standard bright-field microscopy; the latter uses fluorophores, fluorescent markers which allow for staining of multiple targets (e.g., collagen for basement membrane and skin annexes). Indirect immunofluorescence can also employ confocal microscopy to provide depth analysis of samples and observe them in their full range.

IENFD stands as the strongest bioptic parameter in determining the status of small nerve fibers, as demonstrated in the most recent SFN criteria, where distal leg IENFD reduction constitutes a separate criterion when compared to other techniques to assess small nerve fiber status [13]. Reference values have been provided, adjusted for sex and age, for both bright-field immunohistochemistry [3] and indirect immunofluorescence [14].

While not incorporated in criteria, other aspects have been explored in skin biopsy. Most notably, morphological alterations of small nerve fibers, such as branching and swelling, have long been recognized as constituting early stages of neuropathy when IENFD has still not been affected, as was the case for painful symptomatology linked to diabetes mellitus [15]. Moreover, other measures such as epidermal and dermal nerve fiber length densities have been employed in some cases, while more complex than fiber counting as for IENFD.

Concerning the autonomic nervous system, the exact mechanisms and patterns of sympathetic and parasympathetic skin innervation have not yet been fully elucidated, while indirect immunofluorescence studies have provided valuable new data. The complex geometry underlying the innervation of “tortuous” structures such as blood vessels, sweat glands, and arrectores pilorum muscles makes the quantitative counting of autonomic nervous fibers complicated and still require time-consuming methods, thus not proving convenient so far. While not as strong, qualitative and semi-quantitative analyses, in which a score is attributed by an expert examiner based on the abundance of fibers and architecture of autonomic structures, have been proposed [16].

### 2.2. Ganglionopathies

The role of skin biopsy has not been limited to SFN alone: it has been shown that this method can provide key information in processes like ganglionopathy, in which IENFD from more proximal sites such as the thigh can disclose a non-length-dependent process [17].

Ganglionopathies can be caused by a significantly large array of etiologies, some of which require prompt recognition and management, as is the case of paraneoplastic sensory neuronopathies.

Among various causes, Spinobulbar Muscular Atrophy, i.e., Kennedy Disease, is an inherited form of motor neuron disease in which dorsal root ganglia is characteristically affected, demarcating this entity from the others of the same neurological group. Consequently, skin biopsy has demonstrated a non-length-dependent impairment of small and large nerve fibers in such patients [18].

Skin biopsy has also helped in recognizing peculiar patterns of neuropathy in disorders such as Cerebellar Ataxia, Neuropathy, Vestibular Areflexia Syndrome (CANVAS) [19], a pathology related to RFC1 expansions causing sensory axonal neuropathy and, particularly, chronic cough, which could be related to non-length-dependent damage to laryngeal small nerve fibers.

### 2.3. Hereditary Neuropathies (CMT, HSAN)

Aside from small nerve fibers, skin biopsy can also disclose information about larger, myelinated nerve fibers. It has been shown that immunohistochemistry can reliably detect myelinated endings and mechanoceptors [20], suggesting a potential role of skin tissue as a sensitive biomarker of both large and small neuropathic disease. This was subsequently proven in hereditary Large-Fiber Neuropathies such as Charcot–Marie–Tooth disease (CMT), which actually constitutes a variegate group of both demyelinating and axonal neuropathies with different inheritance patterns. In the most common form of CMT, CMT type 1 A (CMT1A), skin biopsy was able to disclose both macroscopic nerve abnormalities (e.g., shortened internodal length) and abnormalities at the molecular level, such as increased levels of the PMP22 protein, consistent with the etiological duplication of the relative gene in CMT1A. Reduced PMP22 levels were instead seen in the skin tissue of patients with Hereditary Neuropathy with Liability to Pressure Palsy (HNPP), caused by PMP22 gene deletions [21,22].

Hereditary Sensory and Autonomic Neuropathies (HSANs) are instead inherited forms of neuropathies in which skin biopsy detects the complete compromission of small nerve fibers, such as that seen at the distal leg site of HSAN1 patients (Fridman). In the same study, such peculiar abnormalities of skin biopsy suggested that this technique could be used as a sensible and repeatable disease biomarker [23].

### 2.4. Inflammatory Neuropathies

Inflammatory neuropathies are acquired disorders in which autoimmunity leads to demyelination and damage of nerve fibers.

Guillain-Barré syndrome, i.e., Acute Inflammatory Demyelinating Neuropathy (AIDP), is typically viewed as a large fiber polyradiculoneuropathy; however, the disorder characteristically affects the autonomic small nerve fibers, leading to life-threatening complications such as arrhythmias and blood pressure dysregulation. Additionally, pain is another symptom which may be overlooked, also suggesting somatic nerve involvement. Consequently, skin biopsy has been explored in AIDP patients, in which small-fiber impairment could be demonstrated and was shown to correlate with disability grade [24]. Interestingly, skin biopsy showed normal dermal and epidermal innervation in patients with the Acute Motor Axonal Neuropathy (AMAN) form of AIDP, in which motor-point biopsies instead showed intramuscular nerve fiber alterations [25].

Chronic Inflammatory Demyelinating Polyneuropathy (CIDP), the other main entity, is constituted by different forms of disease encompassing motor and sensory impairment, while autonomic involvement is less typical. While skin biopsy has been less explored, an exploratory 2022 study has found that IENFD was similarly reduced in typical and variant forms of CIDP, with moderate correlation to disability scales, suggesting a potential usefulness of extending such a technique in a wider number of patients [26].

The role of skin biopsy in different peripheral neuropathies is summarized in the following table (Table 1):

**Table 1:** Skin biopsy in peripheral neuropathies.

Disease	Application
Small-Fiber Neuropathy	- Diagnosis: evaluation of IENFD at distal leg [13]; - Bright-field immunohistochemistry or indirect immunofluorescence [3]; - Analysis of autonomic skin structures [16]; - Possible markers of pre-neuropathic stages: axonal swelling, increased branching [15]; - Structural assessment alone, does not assess in vivo small nerve function; - Inflammatory: may correlate with disease in AIDP small-fiber impairment [24]; potential utility in CIDP, too [26]; - Inherited: may show peculiar denervation patterns (e.g., total absence of nerve fibers in HSAN) [23].
Large-Fiber Neuropathy	- Diagnosis: assessment of myelinated fibers (e.g., nerve endings) [20]; - Inherited: in CMT, skin biopsy can provide disease markers at both structural and molecular levels [21,22].
Ganglionopathy	- Diagnosis: IENFD altered at both distal (leg) and proximal (thigh) sites without gradient [17]; - Differential diagnosis: may help identify peculiar non-length-dependent disorders, such as Kennedy disease 18, CANVAS [19].

## 3. Metabolic and Vascular Disorders: Fabry Disease, Amyloidosis, CADASIL, and CARASIL

Metabolic, systemic disorders in which pathological deposits are found in the nervous system make for apt targets of skin biopsy as a diagnostic method.

This is certainly the case for Fabry Disease (FD) [27], a lysosomal, X-linked disease in which mutations of the GBA gene encoding for the α-galactosidase enzyme lead to widespread accumulation of globotriaosylceramide-3 (Gb3), particularly affecting the heart, kidney, and nervous system [28].

Among the variegate symptoms and signs of FD, pain is a key manifestation, presenting early in childhood in the classical, full-fledged form of disease [29]. While the pain and autonomic dysfunction phenotype in FD is quite complex and certainly deriving from different pathophysiological mechanisms, a neuropathic component is predominant [30]. Most importantly, neuropathic pain in FD derives from both dorsal root ganglia involvement and the subsequent development of SFN27, thus both being length- and non-length-dependent processes. Skin biopsy then becomes a helpful determinant of small nerve fiber status, which could act as a marker of disease development in mutation carriers.

Apart from small nerve fibers and similarly to biopsies of other organs, skin biopsy can show Gb3 deposits, particularly in blood vessels, arrectores pilorum muscles, and sweat glands. Thus, skin represents an easily accessible organ to assess FD-specific pathology [30].

Amyloidosis is a wide term encompassing disorders characterized by pathological deposition of protein aggregates (amyloids) [31,32]. It is characterized by prominent peripheral nervous system involvement, especially affecting the autonomic component with disabling symptomatology [33].

Similarly to FD, skin biopsy in amyloidosis can disclose small nerve fiber pathology [34], but also pathological amyloid deposition with specific (Congo red) staining advanced techniques such as electronic microscopy [33]. Owing to these advantages, the role of skin biopsy has also been recognized in clinical trials for treatment of different types of amyloidosis [35].

Lastly, examination of pathological deposition in blood vessels via skin biopsy has been paramount in a non-metabolic disease, Cerebral Autosomal Dominant Arteriopathy with Subcortical Infarcts and Leukoencephalopathy (CADASIL), a genetic disorder caused by NOTCH3 mutation and characterized by small-vessel disease leading to early-onset cognitive impairment and stroke [36].

Before widespread genetic analysis was available, diagnosis was supported by electronic microscopy of skin tissue, which shows specific granular osmiophilic bodies in vessels29. More recently, skin biopsy has proven to be an accurate method for detecting Notch3 extracellular domain deposits in vessel walls and may thus represent a potential alternative to brain biopsy [37].

Cutaneous somatic and autonomic fiber involvement with vascular derangement parallels—to some extent—the central manifestations of the disease [38]. Comparable observations have been reported more recently in patients with cerebral autosomal recessive arteriopathy with subcortical infarcts and leukoencephalopathy (CARASIL) [39].

## 4. Synucleinopathies

### 4.1. Introduction

α-synucleinopathies are neurodegenerative disorders characterized by aggregation and deposition of the protein α-synuclein (p-syn) in the nervous system. Ranging from the most common entity, Parkinson’s Disease (PD), to the other diseases, Dementia with Lewy Bodies (DLB), Multiple System Atrophy (MSA), and Pure Autonomic Failure (PAF), synucleinopathies may affect both the central and peripheral nervous system, causing a wide range of motor (parkinsonism) and non-motor (dysautonomia, sleep disorders, neuropsychiatric alterations, cognitive decline) signs and symptoms with an impactful effect on quality of life and survival.

Precise diagnosis of specific synucleinopathic disorders is crucial in clinical practice, as efficacious treatment is available for PD, whereas the other disorders (except for PAF) carry a worse prognosis without significant therapeutic strategies. Nonetheless, identification of these diseases may be impaired by overlapping clinical presentations among synucleinopathies themselves and with other neurodegenerative disorders, including, for instance, Alzheimer’s Disease (AD), whose pathology often coexists with that of synucleinopathies in patients with cognitive decline [40].

Thus, novel techniques are needed to detect synuclein pathology in earlier stages, similar to those being developed in other neurodegenerative disorders such as AD, in which amyloid and tau pathology can be identified via imaging and biological markers, now allowing for early diagnosis even before clinical presentation [41].

Skin tissue offers easy access to the peripheral nervous system: in synucleinopathies, small nerve fibers are also targets of pathological α-syn deposition, which can be identified with different techniques, such as bright-field immunohistochemistry, first proposed in 2010 [42] with low sensitivity in living patients [16] and similar limitations in colocalization of different signals, as discussed above for SFN. Indirect immunofluorescence, first introduced in 2013 by the Bologna skin lab [43], has shown more promising results in identifying pathological synuclein accumulation.

### 4.2. Diagnostic Accuracy and Differential Diagnosis

Over the course of its development, skin biopsy for p-syn identification has shown high sensitivity in detecting synucleinopathies and their prodromal stages, and near-complete specificity in distinguishing them from both healthy controls [43,44,45,46,47] and from other neurodegenerative disorders, such as vascular parkinsonism and tauopathies including Progressive Supranuclear Palsy (PSP)/Corticobasal Degeneration (CBD) and Frontotemporal Dementia (FTD) [48].

Furthermore, skin biopsy has demonstrated potential accuracy in stratifying the clinical variants of synucleinopathy (including MSA, PD, PAF, DLB) based on the peripheral p-syn load and topographic distribution, suggesting distinct pathogenic mechanisms despite phenotypic similarities [46]. For instance, in PD patients, a prevalent deposition in autonomic fibers has been consistently reported [46,49,50,51], including a length-dependent sensory and autonomic neuropathy [46] associated with a rostro-caudal paravertebral gradient of p-syn and more severe cervical skin denervation, associated with clinical disease duration and p-syn deposition [52]. In contrast, MSA showed a greater p-syn burden and a more widespread distribution, with deposits in the somatic fibers of subepidermal plexi of the superficial dermis [50].

Prompted by the need for a specific biomarker, p-syn in Remak non-myelinating Schwann cells (RSCs) has been demonstrated to be the pathological hallmark of MSA, absent in PD/DLB, with a reported sensitivity of 74% [53]. Skin alterations recapitulate disease mechanisms, providing confirmation of previously observed central and peripheral pathologies. Indeed, in MSA patients, α-synuclein immunoreactivity has been demonstrated in glial cytoplasmic inclusions (GCIs) in oligodendrocytes and in Schwann cell (SCCIs) [54,55].

Similar diagnostic challenges arise when comparing patients with MSA and PAF. In this setting, analysis of the autonomic innervation of cutaneous annexes proved useful for the discrimination of patients with comparable disease duration: MSA patients showed preserved autonomic innervation, whereas PAF patients exhibited impaired innervation of dermal annexes and depleted adrenergic fibers adjacent to arteriovenous anastomosis [56]. Compared to MSA and PD, both PAF and DLB showed the highest p-syn burden with widespread involvement of autonomic nerve fibers [16]. In PAF, DLB and PD p-syn load was proportional to the presence of autonomic symptoms, with DLB exhibiting an intermediate degree of autonomic involvement [46].

### 4.3. Comparison with Other Techniques

In the evaluation of early in vivo biomarkers, skin biopsy for p-syn detection in the prodromal stages of synucleinopathy outperformed both skin and cerebrospinal fluid (CSF) real-time quaking-induced conversion (RT-QuIC) in a comparative study conducted in idiopathic RBD patients [56]. Previous evidence suggests promising sensitivity and complete specificity of RT-QuIC in differentiating PD from MSA according to different reactivities of distinct α-synuclein conformational strains; however, the signal from MSA CSF samples was lower than from PD, or even undetectable, whereas seeding activity has been accurately detected across the Lewy body synucleinopathy spectrum (DLB, iRBD, PAF, and PD) [57,58].

### 4.4. Technical Aspects

A wide range of technical differences occur in different laboratories performing indirect immunofluorescence on skin biopsy, with potentially significant effects on the reproducibility and reliability of results [47].

One possible protocol, as followed in our center, consists of performing skin biopsy using a 3 mm punch under sterile conditions and local anesthesia; sutures are generally not required. Skin samples are fixed in cold 4% paraformaldehyde or Zamboni’s fixative (4% paraformaldehyde with picric acid) for a period ranging from 30 min at room temperature to approximately 18 h at 4 °C. Freezing is not required, so samples can be stored at 4 °C [48].

Different skin areas should be biopsied for maximizing the diagnostic yield of cutaneous p-syn detection according to the clinical suspicion: these include cervical (C7) distal leg and thigh [46,48], but also thoracic (Th12) [51] and fingertip [53].

Originally, two skin samples were collected from each site to increase the probability of detecting p-α-syn. Indeed, the number and localization of skin sites from which to collect samples are among the most differing aspects between laboratories: the ideal procedure would optimize diagnostic accuracy with less sampling possible in order to reduce side effects for patients while retaining most utility. Different studies have assessed whether taking two samples at the same sites significantly increases accuracy compared to single sampling, but no significant difference in diagnostic accuracy was found across various synucleinopathies [46,48,49].

Thickness of sample cuts, ranging from 10 to 50 μm, may also be responsible for different p-syn positivity, likely due to the larger tissue volume increasing the likelihood of detecting the discontinuous distribution of p-α-syn deposits [59,60].

Aside from purely qualitative analysis, quantitative analysis could constitute a promising marker of disease activity and progression. Different methods to quantify such a load of misfolded p-α-syn have been described, mainly including the distribution coefficient (DC) (which calculates the p-α-syn location across the various skin sites biopsied), the fiber rate, the number of p-α-syn fibers in relation to the total number of annexes analyzed, or the absolute number of positive p-α-syn fibers [46].

From a technical point of view, different commercially available primary antibodies raised against p-α-syn can been used. The best yield has been seen with monoclonal antibodies from either rabbit or mouse, whereas polyclonal antibodies frequently lead to non-specific signals. As reported in the original Bologna skin lab description, p-α-syn should be co-stained with a neuronal marker, usually against the pan-neuronal protein gene product PGP 9.5, or NCAM expressed in skin glial non-myelinating Schwann cells, i.e., Remak cells [48,53]. The co-staining allows for differentiation of pathological misfolded α-syn from a false signal arising from the background staining. As secondary antibodies, those associated with cyanine dye fluorophores or Alexa Fluor(R) are preferably used, allowing for visualization with a confocal microscope mounting appropriate fluorescent filters. The use of distinct fluorophores enables co-localization studies, by combining different primary antibodies without retrieval methods [48].

The role of skin biopsy in synucleinopathies is summarized in the following table (Table 2):

**Table 2:** Skin biopsy in synucleinopathies.

Disease	
Parkinson’s Disease	- Detection of p-syn deposits, which may be localized (cervical/leg) [52]; - Autonomic adrenergic fibers of cutaneous vessels may be particularly affected [46,49,50,51]; - Detection of small-fiber pathology [50].
Dementia with Lewy Bodies	- More widespread p-syn distribution [46,48]; - Autonomic more than somatic fiber involvement [46,48].
Multiple System Atrophy	- Widespread p-syn distribution [46]; - Somatic more than autonomic fiber pathology [49,50,51]; - Glial cell involvement [53].
Pure Autonomic Failure	- Widespread p-syn distribution [16]; - Diffuse autonomic involvement [16].
Technical aspects	
Skin sites	- Most commonly cervical (C7)—distal leg—thigh, but also thoracic (T12) and fingertip [46,49,50,51].
Number of skin samples	- No significant difference in diagnostic accuracy when comparing 1 (50 μm) vs. 2 (10–15 μm) [46,49].
Thickness of samples	- Higher positivity may be seen with thicker samples [59,60].
Quantitative syn measures	- Measures such as Distribution Coefficient may help differentiate synucleinopathies [46].
Immunofluorescence technique	- Comparable accuracy also using amplification strategies (e.g., biotin-streptavidin) [46,48].

## 5. Conclusions

Skin biopsy is an increasingly employed technique to assess the status of the peripheral nervous system. It provides information on both large and especially small nerve fiber pathologies, in both its somatic and autonomic components. While key for the diagnosis and definition of Small-Fiber Neuropathy, other structures, especially blood vessels, can become the target of deposition of pathological molecules, such as Gb3 for Fabry Disease and p-syn in synucleinopathies.

The potential role of biopsy in the identification of neurological disorders, even in their early phase, makes it a valuable tool for neurodegenerative disorders, in which rapid recognition is warranted with the future aim of developing disease-modifying therapies. Future research must focus on the application of skin biopsy on more variegated cohorts of patients with suspected neurodegenerative disorders and in healthy individuals who might be predisposed to the development of such diseases. In order to promote the diffusion of this technique, development of reliable protocols is needed to provide comparable data across different laboratories. As for sample variability, consensus should be reached concerning the minimum number of skin samples, body sites, and thickness and diameter of samples; as for technique standardization, skin target, optimal techniques, quantitative measures, and antibodies should be further explored.

## Data Availability

No new data were created or analyzed in this study. Data sharing is not applicable.
